# Long-term Survival and Virulence of *Mycobacterium leprae* in Amoebal Cysts

**DOI:** 10.1371/journal.pntd.0003405

**Published:** 2014-12-18

**Authors:** William H. Wheat, Amy L. Casali, Vincent Thomas, John S. Spencer, Ramanuj Lahiri, Diana L. Williams, Gerald E. McDonnell, Mercedes Gonzalez-Juarrero, Patrick J. Brennan, Mary Jackson

**Affiliations:** 1 Mycobacteria Research Laboratories, Department of Microbiology, Immunology and Pathology, Colorado State University, Fort Collins, Colorado, United States of America; 2 STERIS SA R&D, Fontenay-aux-Roses, France; 3 Department of Health & Human Services, HRSA, HSB, National Hansen's Disease Programs, Laboratory Research Branch, Baton Rouge, Louisiana, United States of America; 4 School of Veterinary Medicine, Louisiana State University, Baton Rouge, Louisiana, United States of America; 5 Department of Research and Development, STERIS Corporation, Mentor, Ohio, United States of America; University of California San Diego School of Medicine, United States of America

## Abstract

Leprosy is a curable neglected disease of humans caused by *Mycobacterium leprae* that affects the skin and peripheral nerves and manifests clinically in various forms ranging from self-resolving, tuberculoid leprosy to lepromatous leprosy having significant pathology with ensuing disfiguration disability and social stigma. Despite the global success of multi-drug therapy (MDT), incidences of clinical leprosy have been observed in individuals with no apparent exposure to other cases, suggestive of possible non-human sources of the bacteria. In this study we show that common free-living amoebae (FLA) can phagocytose *M. leprae*, and allow the bacillus to remain viable for up to 8 months within amoebic cysts. Viable bacilli were extracted from separate encysted cocultures comprising three common *Acanthamoeba* spp.: *A. lenticulata*, *A. castellanii*, and *A. polyphaga* and two strains of *Hartmannella vermiformis*. Trophozoites of these common FLA take up *M. leprae* by phagocytosis. *M. leprae* from infected trophozoites induced to encyst for long-term storage of the bacilli emerged viable by assessment of membrane integrity. The majority (80%) of mice that were injected with bacilli extracted from 35 day cocultures of encysted/excysted *A. castellanii* and *A. polyphaga* showed lesion development that was similar to mice challenged with fresh *M. leprae* from passage mice albeit at a slower initial rate. Mice challenged with coculture-extracted bacilli showed evidence of acid-fast bacteria and positive PCR signal for *M. leprae*. These data support the conclusion that *M. leprae* can remain viable long-term in environmentally ubiquitous FLA and retain virulence as assessed in the *nu*/*nu* mouse model. Additionally, this work supports the idea that *M. leprae* might be sustained in the environment between hosts in FLA and such residence in FLA may provide a macrophage-like niche contributing to the higher-than-expected rate of leprosy transmission despite a significant decrease in human reservoirs due to MDT.

## Author Summary

Leprosy is a progressive disease of the skin and nervous system caused by the bacillus, *Mycobacterium leprae*. Implementation of multiple drug therapy (MDT) for leprosy has significantly reduced the global cases of leprosy. Currently, only a few endemic countries remain where relatively high number of cases persists. Despite global reduction of leprosy and the concomitant decrease in human reservoirs, leprosy transmission and incidence have not declined as expected, suggesting a possible extra-human or environmental source of the bacilli. In the current study, we demonstrate that *M. leprae* can survive long-term within cysts of common environmental free-living amoebae. *M. leprae* residing in amoebal cysts for over 30 days remain fully capable of transferring disease to mouse footpads and retain viability phenotypes after several months residence within amoebal cysts. It is hypothesized that these protozoa provide an intracellular refuge for *M. leprae* in environments for which they would otherwise seem ill suited. Traits allowing bacilli to survive in macrophages may likely be acquired via an evolutionary response against predation by amoebae. The results from this work suggest alternative non-human reservoirs for *M. leprae* exist fostering further study to determine the role of amoebae in the transmission of this Mycobacterium to humans.

## Introduction

Human beings have been afflicted by leprosy for over a millennium. Leprosy is a chronic granulomatous infection of skin and peripheral nerves caused by the bacillus *Mycobacterium leprae*. The bacilli are slow growing obligate intracellular organisms trophic for macrophages, dendritic cells (DC) and Schwann cells in peripheral nerves. The scientific community has reached a generally accepted consensus that *M. leprae* is principally a parasite of humans and is spread primarily thereby [1]. In addition, there have been autochthonous cases of leprosy among native-born Americans in the southern region of the United States with no prior history of foreign exposure. In the same regions, wild armadillos are infected with *M. leprae*. A unique *M. leprae* genotype had been found in the majority of armadillos that was identical to U.S. patients who resided in areas where exposure to armadillo-born *M. leprae* was possible [2]. This is highly suggestive of the fact that armadillos are a significant natural reservoir for the bacilli and, leprosy might be a zoonosis in the these areas. There has also been a substantial history of studies, anecdotal evidence, rationalizations and opinions that argue in favor of additional non-human sources of the bacillus [3]. What is more intriguing is that, despite many years of using multidrug therapy (MDT) resulting in a significant reduction in disease prevalence, transmission remains stubbornly high implicating among other issues, ineffective detection of early infection, case reporting deficiencies or a lack of a thorough examination of potential environmental sources of the bacillus [3], [4].

*M. leprae* is an extremely fastidious organism that, despite over 100 years of endeavor, has not been successfully cultured in artificial medium [5]. It is, thus, classified as an obligate intracellular organism with an evolutionarily minimized genome that is believed to have constrained its growth to the intracellular niche. With such a stringent requirement for survival, several questions remain as to how the bacillus remains viable and infectious between human hosts. Are there environmental elements that are capable of sustaining viable *M. leprae* for long periods or are these bacilli dependent on close-quartered conditions necessary for aerosol transmission from human to human? is *M. leprae* harbored in soil and water niches? is the bacillus sheltered and capable of surviving intracellularly in ubiquitous protozoa such as free-living amoebae (FLA) that provide similar micro-niches as human macrophages? Evidence of an environmentally sustainable entity for *M. leprae* would certainly explain, in part, the apparent lack of reduction of the rate of transmission of leprosy in spite of successful MDT [6], [7].

The nature of the relationship between most intracellular organisms and host FLA is currently not defined. The terms “endosymbionts”, or “symbionts” fail to adequately describe these complicated interactions. It is currently proposed to define intracellular microorganisms that associate with FLA without any known directional host/bacterial benefit as “endocytobionts” [7] [8] [9]. Over the past three decades, numerous studies have reported that microorganisms can survive as endocytobionts in FLA. It was reported in 1980 that *Acanthamoeba* harbored *Legionella pneumophila* and that the bacterium resisted phagosome-lysosome fusion and multiplies within the amoebae [8], [9]. This latter work implicated infected amoebae as a source of Legionnaire's Disease. Additionally, there are numerous reports describing infection of *Acanthamoeba* FLA with both pathogenic and environmental mycobacteria such as *M. avium* subsp. *paratuberculosis*, *M. avium-intracellulare*, and *M. bovis* [10]–[13]. In a study involving hospital networks, FLA such as *A. polyphaga*, and *Hartmannella vermiformis* were associated with many species of mycobacteria in water specimens including *M. gordonae*, *M. xenopi*, *M. avium* and *M. kansasii* subtype 1 lending to much circumstantial speculation regarding the means to which mycobacteria have adapted to environmental persistence [14], [15].

The evolutionary response to amoebal predation is the acquisition of traits that confer resistance to digestion in food vacuoles of amoebae [16]. Many *Mycobacterium* species survive and even thrive intracellularly in protozoa [16], [17]. As has been known for many years, mycobacteria have a rich hydrophobic cell wall and, as such, lend themselves quite well to attachment to cellular surfaces and are efficiently phagocytized by macrophages [18] and protozoa [19]. Many elements of the mycobacterial cell wall contribute to efficiently enable an active entry of the bacterium into phagocytes [20] [21] [22]. Furthermore, protozoa possess the remarkable ability to transform into cysts protecting them from harmful and often times rapidly fluctuating environmental influences such as extremes in temperature, drought and a spectrum of biocides [23]. Mycobacteria, in turn, can use the nutrients of protozoa as a food source and their intracellular life offers protection against the potentially harmful extracellular milieu. This poses the interesting question as to whether amoebae provide an environmental niche simply for persistence or are a selective proving ground enhancing virulence. Additionally, the dual lifestyles of amoebae (trophozoite vs. cyst) likely provides a survival niche to fragile, fastidious microbes such as *M. leprae* when the bacillus is subjected to relatively harsh environments such as those between hosts.

Few studies have investigated whether mycobacteria infect amoebae in their natural environment. Thus, an inherent resistance to predation by amoebae likely has important consequences since bacteria that infect and evade amoebal digestion might exploit these traits to enter and resist destruction within macrophages or dendritic cells (DCs) thus thwarting or altering innate immune responses [16], [24]. FLA are environmentally ubiquitous and most are non-pathogenic to immune-competent humans. Delivery of pathogenic mycobacteria within non-pathogenic amoebae to cells of the innate immune system will likely elicit alternative host immune response in comparison to that generated against the *Mycobacterium* alone. This endocytobionic relationship between the somewhat weakly pathogenic bacteria and ubiquitous amoebae and the potential to aid transmission to susceptible host is of great concern to human, animal and ecosystem health.

In the present study we show that *M. leprae* remains viable up to 8 months as determined by the accepted criteria of assessment of membrane integrity by viability staining in 3 species of *Acanthamoeba* (*A. lenticulata*, *A. castellanii* and *A. polyphaga*) and 2 strains of *Hartmannella vermiformis*. Additionally, *M. leprae* extracted from cocultures of *A. castellanii* and *A. polyphaga* that were induced to encyst with the phagocytosed bacilli for 35 days remained viable causing infections and *M. leprae* proliferation in *Foxn1^nu^/Foxn1^nu^* (*nu/nu*) mouse footpads (FP). This works shows for the first time that cysts from amoebae representing species from both *Acanthamoeba* and *Hartmannella* genera are capable of supporting the viability of *M. leprae*, a bacillus so fastidious that it has never been successfully cultivated axenically. The implications of this work relate to the environmental sustainability of *M. leprae* in the context of persistent transmission despite a vastly reduced human reservoir of infection.

## Methods

### Ethics statement

All mouse work was conducted according to relevant U.S. and international guidelines. The procedures for isoflurane anesthesia, infection of *nu/nu* mouse FPs with *M. leprae* and fine needle aspirate (FNA) biopsy are Institutional Animal Care and Use Committees (IACUC)-approved protocols (protocol # 12-3613A and 11-3037A) that are approved/renewed yearly by an institutional review board of certified veterinarians and selected faculty. The mice are monitored twice weekly by trained animal laboratory technicians employed by our Laboratory Animal Resources (LAR) center. Any maladies, whether directly, indirectly or unrelated to the protocol are reported immediately to both the attending veterinarian and the PI (WHW) holding the approved protocol. The committee is in compliance with the U.S. Public Health Service Policy on Humane Care and Use of Laboratory Animals.

### Amoebae strains and culture

Stocks of axenic *Acanthamoeba lenticulata* ATCC 30841, *Acanthamoeba castellanii* ATCC 30232, *Acanthamoeba polyphaga* CCAP 1501/18, *Hartmannella vermiformis* ATCC 50237 and *Hartmannella vermiformis* CHUV 172 were obtained from the American Type Culture Collection (Manassas, VA) and STERIS SA R&D Fontenay-aux-Roses, France. Amoebae stocks were derived from several sources as diverse as ATCC and hospital and city water supplies and were cultivated to axenic stocks using standardized methods [14], [25]. *Acanthamoeba* trophozoites were axenically maintained in culture in 1X PYG medium which consists of Page's amoebae saline (PAS) [60mg NaCl, 2mg MgSO4·7H2O, 68mg KH_2_PO_4_, 71mg NaHPO_4_ and 2 mg CaCl_2_ in 500 ml dH_2_O (pH = 6.9)] to which 1/10 volume of 10XPYG solution [50 g Proteose Peptone (Difco); 5 g yeast extract (Difco); 2.45 g MgSO_4_·7H_2_O; 2.5 g Sodium citrate·2H_2_O; 0.05 g ammonium iron sulfate (NH_4_)_2_Fe(SO_4_)_2_·6H_2_O; 0.85 g KH_2_PO_4_; 0.89 g Na_2_HPO_4_·7H_2_O; 22.5 g α-D-glucose; 0.295 g CaCl_2_ in 250 ml dH_2_O] was added [26] [14]. *Hartmannella* trophozoites were cultured in modified PYNFH (ATCC medium 1034) medium.

### Infection of amoebae with *M. leprae* and extraction of intracellular bacilli

Viable *M. leprae* was obtained from the National Hanson's Disease Programs, Baton Rouge, LA. Trophozoite monolayers of *A. lenticulata*, *A. castellanii*, and *A. polyphaga*, were maintained at 28°C and passaged in 1X PYG. *H. vermiformis* str. ATCC 50237, and *H vermiformis* str. 172 were maintained at 28°C and passaged in PYNFH medium. Amoebae were infected with viable *M. leprae* at a bacilli:amoebae ratio of 5–10∶1 and incubated for 48 hr at 32°C. Extracellular bacilli were removed by centrifugation at 600x*g* and washing the amoebae pellet in HBSS (Hank's Balanced Salt solution) 3 times. For some smaller scale experiments (e.g., for phagocytosis assays), infections were carried out at M.O.I. of between 1 and 100 as well as some cocultures kept at 4°C and aliquots withdrawn every hour to determine adsorption of PHK26-labeled bacilli (see below) to FLA by flow cytometry. FLA- containing *M. leprae* were induced to encyst by pelleting the cultures and subsequently suspending in encystment buffer (0.1M KCl, 0.02M Tris-HCl pH 8.0, 8 mM MgSO_4_, 0.4 mM CaCl_2_ and 1mM NaHCO_3_). Intracellular *M. leprae* was extracted from amoebae cysts maintained at 32°C at various times (one week, two weeks, 35 days, 45 days, 3 months and ≥6 months). Prior to extraction of bacilli, long-term encysted cocultures were induced to transform back to trophozoites in complete growth media at each of the above time points. Bacilli extracted from excysted trophozoites by suspending the pellet in 100 µl of sterile PBS containing 0.5% SDS, vigorously vortexing and washing three times with PBS were then processed for viability using BacLight staining procedure (Molecular Probes; Life Technologies, Grand Island, NY), fluorescence microscopy or injection into mouse FPs.

### Injection of FPs with *M. leprae* extracted from amoebae cocultures

Athymic FoxN1*^nu^*/FoxN1*^nu^* (designated as “*nu/nu*” throughout this manuscript) mice, five in each group, were challenged in the plantar surface of the left hind foot with *M. leprae* bacilli extracted from *A. castellanii* or *A. polyphaga* cysts as described [27]. Mice were injected a total of 3 times every other week for one month. All bacilli used in experimental FP injections were extracted from 35-day encysted *A. castellanii* or *A. polyphaga* cocultures. This three-time injection scheme was performed because the bacillary yield from the extraction process seemed rather low and would ensure a relatively timely appearance of FP induration. The inocula were estimated based on direct counting of bacilli. [28].

### Fine needle aspirate of nu/nu mouse FPs

Mice were anesthetized by inhalation of 5% isoflurane. Once fully anesthetized, infected mouse FPs were aspirated using a 0.5 cm, 23-ga needle syringe inserted subcutaneously into the infected area of the FP. Portions of the samples were prepared for microscopy by acid-fast staining or for nucleic acid extraction for PCR analysis. This procedure was performed monthly for 6 months.

### Nude mouse footpad derived *M. leprae*

The Thai-53 isolate of *M. leprae* was maintained in the footpads of athymic *nu*/*nu* mice infected for approximately 6 months, and then harvested as described previously [27]. Extracted bacilli were washed by repeated (2X) suspension and centrifugation in RPMI-1640 (Gibco) containing 10% fetal bovine serum ((FBS) Gibco). Bacilli were enumerated by direct counting according to Shepard's method [29]. *M. leprae* suspensions were purified by NaOH treatment as described [27]. Briefly, 1 X10^9^ fresh *M. leprae* were suspended in 1 ml of 0.1N NaOH and incubated for 3 minutes at room temperature to remove animal tissue. The bacteria were subsequently washed 3X in Hanks Balanced Salt solution (HBSS) and suspended in a final volume of appropriate medium. Freshly harvested viable bacilli were consistently used in experiments within 24–32 hr of harvest. *nu*/*nu* mice, five in each group, were challenged in the plantar surface of the left hind foot with 10^7^ *M. leprae* harvested from passage animal FP as described [27].

### *M. leprae* from armadillo tissue

Concentrates of *M. leprae* are separated from infected livers or spleens of 9-banded armadillos (*Dasypus novemcinctus*). The tissues were collected aseptically and kept frozen at −80°C. Briefly, the procedure for preparation of *M. leprae* has been described earlier [30], [31] and is carried out at 0 to 2°C. The tissue is homogenized and separated by density gradient centrifugation in sucrose and KCl. The bacilli are disrupted by ultrasonic oscillation. Tissues were treated with trypsin, chymotrysin, collagenase and 0.1N NaOH to remove any host tissue. The bacilli are intact but presumed nonviable due to prolonged storage of the tissue at below freezing temperatures.

### Staining of *M. leprae* with vital red fluorescent dye PKH26

For conventional and confocal microscopy and phagocytosis assays *M. leprae* freshly harvested from FPs were stained with the vital fluorescent red PKH26 dye (Sigma-Aldrich) following the manufacturer's protocol. Briefly, bacilli were stained for 3 minutes at RT in a 1∶250 dilution of dye in Diluent-C (Sigma-Aldrich). The staining suspension was washed three times in PYG containing 5% bovine serum albumin. Bacilli were counted by fluorescence microscopy by averaging several fields counted using a hemocytometer.

### Phagocytosis assay for *M. leprae* and amoebae

Healthy actively dividing amoebae trophozoite cultures were seeded in 6-well plates at 3×10^6^/ml containing appropriate growth medium (above). Amoebae were infected with viable *M. leprae*, or *M. leprae* isolated from armadillo tissues (presumed non-viable) that were first stained with the red fluorescent vital PKH26 membrane dye. Triplicate *M. leprae*-infected amoebae cultures were prepared at bacilli∶amoebae ratios of 1∶1, 5∶1, 10∶1, 50∶1 and 100∶1. Cultures were maintained either in a humidified incubator at 32°C or in a cold room at 4°C. Amoebae were harvested at 0 time (at the time of *M. leprae* challenge), 2 hr, 3 hr, 4 hr, 5 hr and 6 hr post-infection, washed twice to remove extracellular *M. leprae* and suspended in 300 µl of FACS buffer (PBS +1%BSA) prior to analysis by flow cytometry using a Becton Dickinson FACS Cantos II instrument. Results were gathered from gates of uniform size as determined from uninfected samples. The resulting mean fluorescence intensities (MFI) were acquired as one-color histograms and increases in MFI were plotted against time using GraphPad Prism software. Results are shown as the average MFI of triplicate cultures.

### BacLight staining fluorescent viability staining of *M. leprae*

The viability of *M. leprae* was determined by assessing membrane integrity using the LIVE/DEAD BacLight bacterial viability kit (Molecular Probes; Life Technologies, Grand Island, NY). Bacilli extracted from amoebae cyst cultures were washed in normal saline (0.90% NaCl w/v) and incubated for 15 min at RT with a final concentration of 1.67 mM Syto9 and 18.3 mM propidium iodide (PI). The bacilli were subsequently washed twice in normal saline (NS) and the pellet was suspended in 25 µl of NS and 5 µl was spotted on a glass slide and mounted on a #1.5 cover glass using BacLight mounting oil. The dead and live bacteria were assessed by direct observation of fluorescent red (PI^+^) and green (Syto9^+^) bacilli respectively under a fluorescence microscope using appropriate single bandpass filter sets [FITC filter (480 nm excitation/500 nm emission for Syto9); TRITC filter (488 excitation/653 excitation for PI)]. In cases of nuclear staining of amoebae or mouse tissues, a DAPI filter (358 nm excitation/461emission) was utilized.

### Staining of infected amoebae auramine/rhodamine

Auramine/rhodamine was used to visualize acid-fast bacilli (such as mycobacteria) using fluorescence microscopy. Staining was performed as is routine in the laboratory. Briefly, aliquots of *M. leprae*-infected or uninfected cysts or trophozoites were transferred to microscope slides and heated to 78°C for 30 min. Slides were then stained for 30 min at RT with auramine/rhodamine (Becton Dickenson, Franklin Lakes, NJ). Slides were rinsed with acidified-alcohol (5% HCl/70% isopropanol) followed by staining with Hematoxylin QS (Vector Laboratories, Burlingame, CA) for 5 sec. Slides were rinsed with dH_2_O and stained with DAPI (200 µg/ml) for 20 min. The slide were washed with dH_2_O and mounted to cover glasses with Prolong Gold (Life Technologies, Grand Island, NY). Slides were visualized using a fluorescence microscope within 24 hr of preparation/staining.

### Fluorescence and confocal microscopy

Both fluorescence and confocal microscopes were used to visualize extracted and internalized *M. leprae* by all amoebae spp. studied. Fluorescence microscopy was performed with an Olympus IX71 microscope (Center Valley, PA) using Retiga 2000R (Qimaging, Surrey, BC, Canada) and Qcolor3 (Olympus) cameras. Qimaging and Slidebook software (Intelligent Imaging Innovations, Inc., Denver, CO) were used for image acquisition and analysis on a Macintosh G5 dual processor computer (Apple Computer, Cupertino, CA). Confocal microscopy was performed on a Zeiss LSM 510 confocal microscope. To determine the spatial occupancy of *M. leprae* within amoebae, serial optical sections were imaged of infected amoebae and were taken at 0.2 nm intervals using a 514 nm excitation laser and 560±20 nm emission filters.

### PCR analysis of *M. leprae* infection

Nucleic acid extraction from amoebae cocultures from fine needle aspirate tissue samples was performed using the Qiagen DNeasy kit and PCR amplification was performed on 50 ng extracted DNA using primers that amplify the *M. leprae*-specific repetitive element (RLEP) [32]. Amplified PCR samples positive for the presence of *M. leprae* produced a 129 bp product.

### Real-time TaqMan PCR analysis to quantify *M. leprae* in FPs

To assess growth and counting efficiency of *M. leprae* in FPs real-time TaqMan PCR assays were performed. *M. leprae* genomic DNA was obtained from FP tissue homogenates as described elsewhere [33]. Briefly, 200 µl aliquots of tissue homogenates were subject to 3 freeze/thaw cycles, and proteinase K was added to 10 mg/ml and the sample were incubated at 56°C for 2 hrs. The genomic DNA was processed and purified using the DNeasy Kit (Qiagen, Inc, Valencia, CA) according to the manufacturer's directions. Molecular enumeration of *M. leprae* was determined using purified DNA fractions from each specimen via TacMan technology using primers and a probe for a common region of the RLEP family of dispersed repeats in *M. leprae* as previously described [33] [32]. The specific sequences of the primers and probe have been described elsewhere [34]. All reagents used in the TaqMan assay were recommended by the manufacturer (PE Applied Biosystems), including AmpErase UNG enzyme and AmpliTaq Gold DNA polymerase. PCR cycling conditions were 40 cycles with 60°C annealing/extension temperature for 60 seconds and 95°C denaturing temperature for 15 seconds. PCR and data analyses were performed on a 7300 RealTime PCR System (Applied Biosystems, Foster City, CA).

## Results

### Phagocytosis of *M. leprae* by three species of *Acanthamoeba* and two strains of *Hartmannella vermiformis*

**Figure 1:**
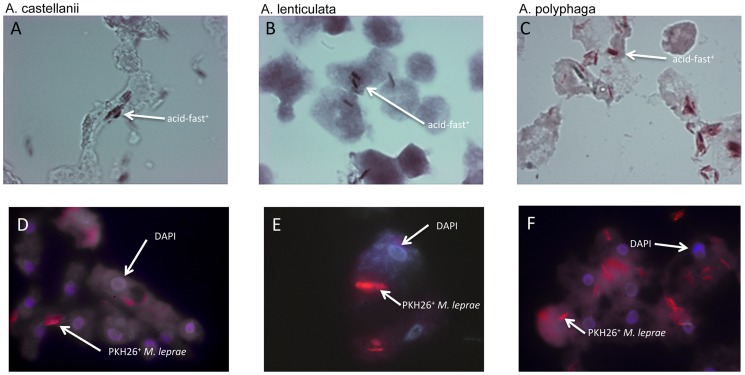
Amoebae were cultured for 16 hr at 32°C at an M.O.I. of 10 with live fluorescently labeled (PKH26) *M leprae* freshly isolated from mouse footpads. Cocultures were washed with 1/10 PYG and centrifuged at slow speed 3X to remove free *M. leprae*. Samples were treated with 1% paraformaldehyde in PBS (pH = 7.00) for 15 prior to mounting on slides. Microscopy slides were acid-fast stained and mounted with coverslips using a DAPI-containing medium to observe amoebae nuclei. Samples were viewed under visible light (panels A, B, and C) or TRITC-filtered fluorescent light to reveal fluorescent *M. leprae* within amoebae: *A. castellanii* (panel D), *A. lenticulata* (panel E) and *A. polyphaga* (panel F). Arrows indicate enveloped acid-fast bacilli (panels A, B and C) or fluorescent *M. leprae* and/or amoebae nuclei (panels D, E and F).

To show that amoebae are capable of phagocytosing *M. leprae* bacilli, we infected amoebae trophozoite cultures with *M. leprae* at an M.O.I. of 5. To facilitate infection, the trophozoite cultures were shifted from optimized medium to 1/10 the optimum nutrient concentration allowing for a parallel shift from a primarily pinocytotic nutrient acquisition mode to a macro-phagocytic mode that effectively optimizes the trophozoites to take up the bacilli [35]. Fig. 1 shows light microscopy of acid-fast staining of *M. leprae* in cocultures established by infecting three species of *Acanthamoeba* with freshly harvested viable *M. leprae.* Greater than 95% of the amoebae were observed to be internally occupied (Fig. 1; Panels A–C) with at least one acid-fast bacillus residing in the amoebic trophozoites. At relatively low M.O.I. (1∶1 to 5∶1), ingestion of live *M. leprae* did not exert any observable adverse effect on amoebae that divided normally over several days. At higher M.O.I. (>5∶1), however, the bacterial burden negatively affected the growth of trophozoites and the cocultures showed low-level lysis of amoebae and considerable detachment from plate wells. *M. leprae* bacilli were also readily taken up by two strains of *H. vermiformis* (str. ATCC 50237 and str. 172) as well. Fluorescence microscopy of PKH26-labeled *M. leprae* in cocultures of amoebae that were stained by the DNA-specific dye, DAPI, showed that the bacilli were taken up into areas that were mostly exclusive to nuclear staining indicative of primarily cytoplasmic staining (Fig. 1 D–F).

**Figure 2:**
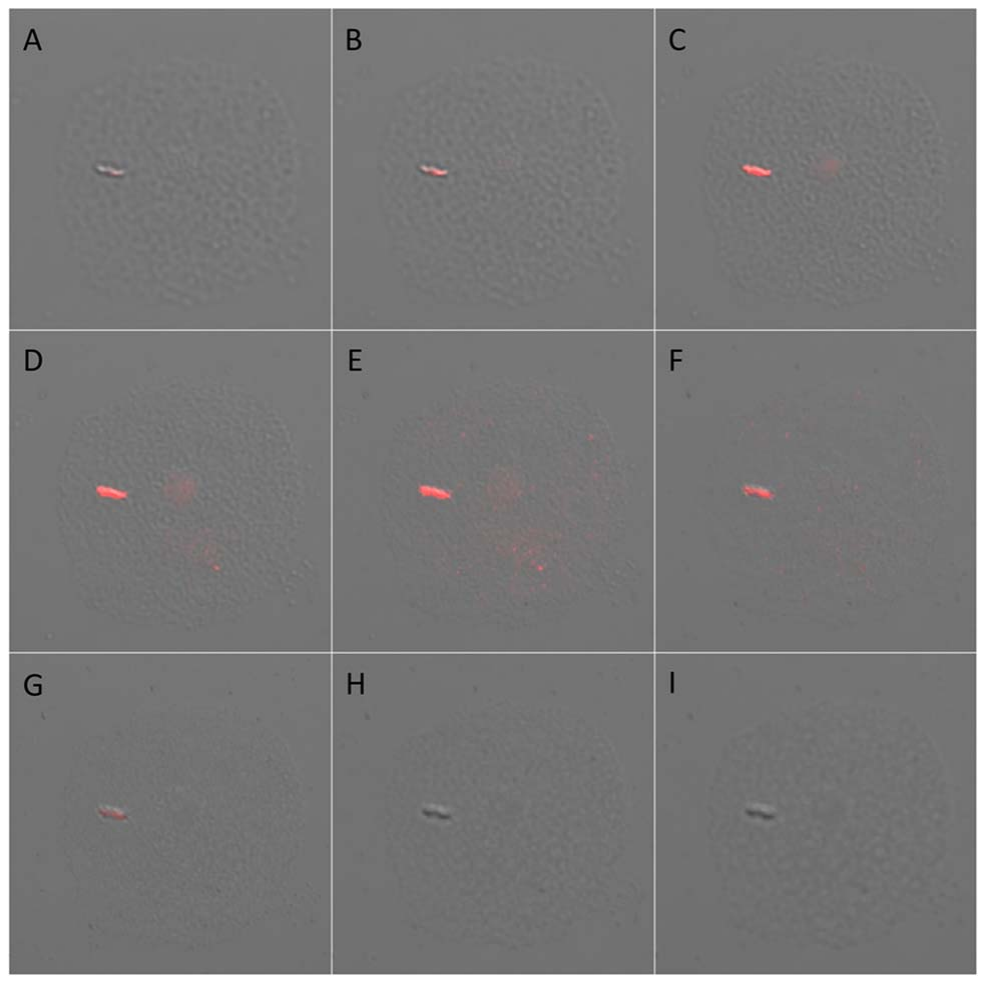
Confocal microscopy (600X) of fluorescent (PKH26) stained live *M. leprae* phagocytosed by *A. polyphaga*. Nine successive focal planes (A–I) from top through the bottom of the amoebae were imaged showing the bacillus to be located internally rather than simply being adsorbed on the protozoan exterior.

In order to determine whether the *M. leprae* bacillus is phagocytosed by amoebae as opposed to being merely adsorbed to the protozoan surface, we prepared cocultures as above and examined the fluorescently labeled bacteria by confocal microscopy following 16 hr of culture (Fig. 2). Confocal microscopy was utilized to resolve the physical location of the *M. leprae* bacilli within infected amoebae at various focal planes. Layered focal resolution of *M. leprae*-infected *A. polyphaga* showed that the best optical and fluorescent resolution of the bacilli was well within the interior of the amoebae suggesting that the bacilli resided within the amoebae interior as opposed to their external surface. Similar resolution was obtained for *A. castellanii*, *A. lenticulata* as well as both strains of *H. vermiformis*.

### Endocytosed *M. leprae* reside primarily in acid-rich regions of amoebae

**Figure 3:**
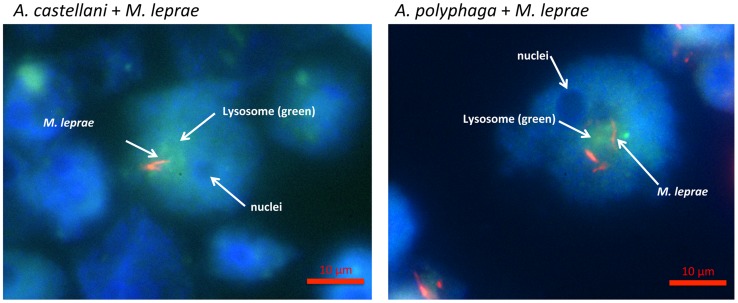
*M. leprae* bacilli co-localizes with acid-rich regions of amoebae cytoplasm. Axenic cultures of *A. castellanii* and *A. polyphaga* were infected with fresh viable *M. leprae* from *nu/nu* mouse footpads for 16 hr at 32°C in 1/10 PYG followed by pulsing with 100 µM Lysotracker Green DND for 2 hours.

Amoebae were cultured with live PKH26-stained *M. leprae* in appropriate amoebae medium for 16 hr to allow for complete envelopment of the bacilli. Following uptake of *M. leprae*, the cultures were pulsed at 32°C for 2 hr with 100 mM Lysotracker Green-DND-26 (Molecular Probes, Life Technologies, Grand Island, NY) in order to fluorescently stain the acid-rich organelles such as lysosomes residing within the amoebic cytoplasm. Fig. 3 shows that the fluorescently labeled *M. leprae* infecting either *A. castellanii or A. polyphaga* resided primarily within acid-rich organelles (i.e. lysosomal compartments) of amoebae similar to what is observed in macrophages, DCs and Schwann cells. The bacilli were similarly located within the cytoplasmic regions of *A. castellanii*, *A. lenticulata*, *H. vermiformis* str. ATCC 50237 and *H. vermiformis* str. *172.*

### Optimal uptake of the *M. leprae* bacilli depends on both physiological temperature optima and the viability of the infecting bacilli

**Figure 4:**
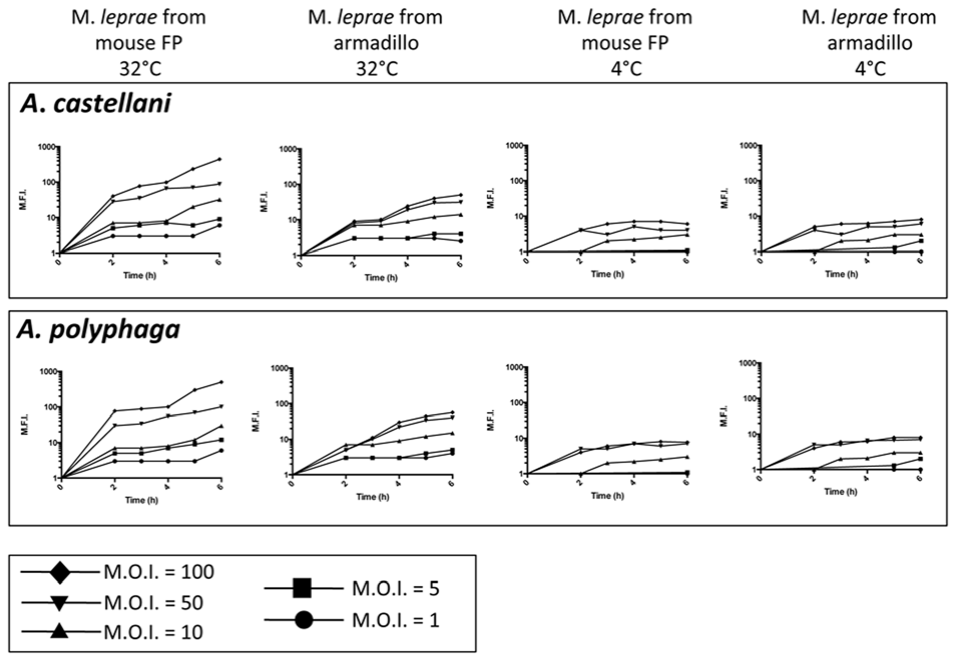
Axenic cultures of *A. castellanii*, and *A. polyphaga*, were infected with *M. leprae* isolated from either *nu/nu* footpads or armadillo tissue at various M.O.I. (1∶100 (black diamonds), 1∶50 (black inverted triangles), 1∶10 (black upright triangles), 1∶5 (black squares) and 1∶1 (black circles) [amoebae:*M.lepra*e]) in 1/10 PYG at either 32°C or 4°C. Aliquots were taken at the time of infection and each hr after 2 hr of incubation and analyzed by flow cytometry. Prior to flow cytometric analysis the aliquots were washed with 1/10 PYG and centrifuged 3X at 600×g to pellet and remove any cell-free bacilli. Samples were analyzed by flow cytometry and the mean fluorescence intensity (M.F.I.) was plotted per unit time (hr) in culture.

To determine whether uptake of *M. leprae* by amoebae requires metabolic viability of either amoebae and/or bacilli, phagocytosis assays were performed that measure the extent of uptake of PKH26-labeled *M. leprae* by *A. lenticulata*, *A. castellanii* or *A. polyphaga* and the two strains of *H. vermiformis*. Fluorescently-labeled *M. leprae* were introduced to actively growing amoebae trophozoites and the extent of acquisition of fluorescence over a 6 hr period was determined by flow cytometry as measured by gain of mean fluorescence intensity (M.F.I.) by the amoebae (Fig. 4). Trial assays showed that the maximal extent of M.F.I. was routinely achieved at 6 hr post-challenge for all amoebae tested. Fig. 4 indicates such for infected axenic cultures of *A. castellanii* and *A. polyphaga* (and all amoebae tested (S1 Fig.). Infections of a M.O.I. greater than 100 proved detrimental to the amoebae and demonstrated lower overall M.F.I. per unit time. The acquisition of red fluorescence by the amoebae as a function of time at 32°C is shown in Fig. 4. In all cases, the best acquisition of red M.F.I. occurred if the temperature was 32°C and the infecting *M. leprae* were viable (i.e. from passaged *nu*/*nu* mouse FP). *M. leprae* freshly isolated from mouse FP and deemed viable by both radiorespirometry and viability staining (>90% viable) proved to be optimally phagocytosed. *M. leprae* harvested from armadillo tissues, has very low or no viability, [30], [31]. Armadillo-derived *M. leprae* was not capable of transferring to amoebae the level of red fluorescence achieved by their mouse FP extracted counterparts, achieving only approx. 10% of the maximal M.F.I. (Fig. 4). In addition, assays performed at the sub-physiological temperature of 4°C showed that the amoebae achieved only about 1% of the M.F.I. of viable *M. leprae* and 10% of *M. leprae* from armadillo tissue when compared to their respective counterparts at 32°C (Fig. 4). Furthermore, most surface adsorption of the fluorescent *M. leprae* to the amoebae at 4°C was removed by rigorous washing of the cells. Collectively, these data suggest that uptake and internalization of *M. leprae* by amoebae optimally requires active amoebae metabolism driving phagocytosis of viable bacilli.

#### Persistence and viability of *M. leprae* inside amoebic cysts

**Figure 5:**
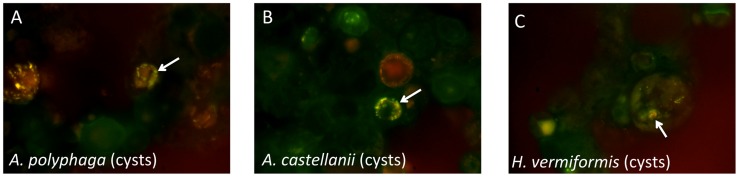
Auramine/rhodamine staining of 6 month cocultures of *M. leprae* and amoebae that were induced to encyst and remained at 32°C under encystment conditions. Arrows indicate acid-fast^+^ staining peripherally in amoebic cysts.

Staining of long-term (6 months) encysted *M. leprae*/amoebae cocultures (maintained in encystment buffer at 32°C) with auramine/rhodamine for acid-fast organisms confirmed the presence of bacilli in *A. polyphaga, A. castellanii* and *H. vermiformis* cysts. Many bacilli were distributed peripherally within the cysts similar to what has been observed in *A. polyphaga* cysts containing *M. avium* subsp. *paratuberculosis* [10] (Fig. 5). Cysts remained evident after 6 months without significant numerical decline or apparent integrity for all of the *Acanthamoeba* and *Hartmannella* cocultures. Interestingly, intact auramine/rhodamine stained bacilli in long-term cocultures were observed to be somewhat distorted (more rounded as opposed to rod-shaped) as has been observed in other mycobacteria-amoebae systems [10], [11], [16]. These data support the hypothesis that *M. leprae* bacilli may remain intact for relatively long periods while residing in amoebic cysts.

**Figure 6:**
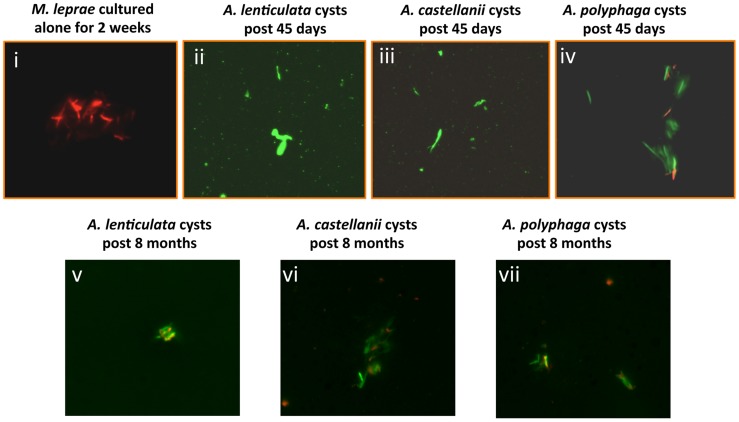
Bacilli extracted from 45 day or 8-month cocultures of amoebal cysts and maintained membrane integrity by preferentially staining with Syto9 (green) and excluding propidium iodide (red) staining in the BacLight LIVE/DEAD assay system. Trophozoites were infected with viable *M. leprae* at an M.O.I. of 5. 48 hours in 1/10 PYG, the cultures were washed and placed in a minimal encystment medium to form cysts. Cultures were maintained at 32°C in a humidified incubator for 45 days (Panels i–vi) or 8 months (Panels v–vii) then transferred to nutrient medium (PYG) for 48 hr and the bacilli from emergent trophozoites were extracted with 0.5% SDS. Samples were examined by fluorescence microscopy. Panel i, *M. leprae* cultured axenically for 2 weeks, Panel ii, bacilli extracted from 45-day cultures with *A. lenticulata*; Panel iii, *A. castellanii*; Panel iv, *A. polyphaga*; Panel iv. Panels v–vii, bacilli extracted from 8-month cocultures of *A. lenticulata*, v, *A. castellanii*, vi and *A. polyphaga*, vii.

*M. leprae* cannot be cultured and thus the assessment of its viability is, at best, cumbersome and remains a major obstacle preventing many avenues of investigation. Indeed, positive staining with auramine/rhodamine is not definitive proof of viability. Recent work has shown a good correlation between the metabolic activity/viability of the leprosy bacilli and their membrane integrity [36]. The use of a two-color, Syto9 and propidium iodide, fluorescence assay which assesses membrane damage in individual bacilli has proven to be a reliable, rapid and direct viability assay for *M. leprae* bacilli that correlates to metabolic-activity data gathered by radiorespirometry analysis [36]. We chose to employ this staining method to assess the viability of *M. leprae* extracted from amoebic cysts that had remained in cultures for up to 8 months. Fig. 6 shows that bacilli extracted from 45 day-old cocultures (panels ii–iv) or from cocultures maintained for 8 months (panels v–vii) were virtually all stained green by Syto9 and mostly excluded the red propidium iodide stain indicating membrane integrity and thus, most likely, viable. As expected, *M. leprae* cultures maintained alone in PYNFH and/or PYG amoebae medium for two weeks mostly stained red with propidium iodide indicating substantial loss of viability (Fig. 6, panel i).

**Figure 7:**
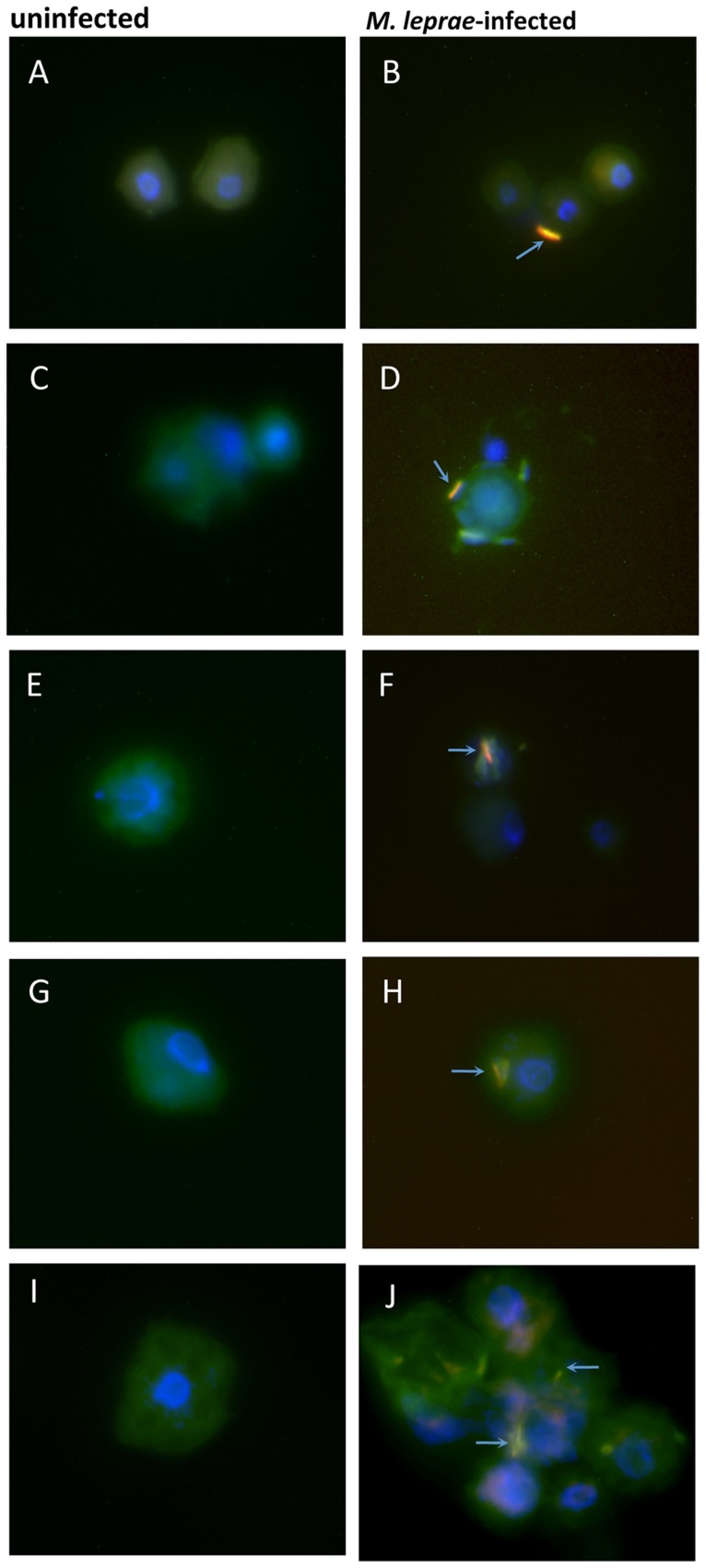
Acid-fast bacilli (arrows) are evident in 35-day old cyst cocultures that were induced to excyst as trophozoites. Cultures of axenic amoebae or *M. leprae*-infected amoebae were infected at an M.O.I. of 5 and incubated for 33 days in encystment buffer followed by 48 hr induction to excyst in 1 X PYG for *Acanthamoeba* or modified PYNFH (ATCC medium 1034) medium for *Hartmannella*. Panels **A**, uninfected *A. lenticulata*; **B**, infected *A. lenticulata*; **C**, uninfected *A. castellanii*; **D**, infected *A. castellanii*; **E**, uninfected *A. polyphaga*; **F**, infected *A. polyphaga*; **G**, uninfected *H*. *vermiformis* str. ATCC; **H**, infected *H*. *vermiformis* str. ATCC 50237; **I**, uninfected *H*. *vermiformis* str. 172 and **J**, infected *H*. *vermiformis* str. 172. Slides were prepared for fluorescence microscopy as described and stained with DAPI for amoebae nuclei, with auramine/rhodamine for acid-fast bacilli (orange) and with Lysotracker Green-DND-26 for visualization of acid-rich cytoplasmic regions of amoebae (green).

Samples of 35 day-old *M. leprae*/cyst cocultures (*A. lenticulata*, *A. castellanii*, *A. polyphaga*, *H. vermiformis* ATCC 50237 and *H. vermiformis* 172) that were allowed to excyst were also metabolically labeled at 32°C with Lysotracker Green and subsequently stained with auramine/rhodamine and DAPI as described above. The samples were then assessed for the extent of acid-fast organisms by fluorescence microscopy. Fig. 7 (panels A–J) compares uninfected vs. *M. leprae*-infected emergent trophozoites for all five of the amoebae genera/strain cocultures and shows that auramine/rhodamine^+^ bacilli (arrows) were present within the cytoplasm of all emergent trophozoites. Interestingly, in the case of the *A. castellanii* cocultures, most of the acid-fast bacilli were observed to be peripherally distributed in the recently emergent trophozoites (Fig. 7D) indicative of being arranged in such a manner within the cysts. None of the uninfected amoebae cultures showed any evidence of containing acid-fast organisms indicating that the acid-fast bacilli observed in the infected cocultures were solely the result of *M. leprae* infection (Fig. 7, first column).

**Figure 8:**
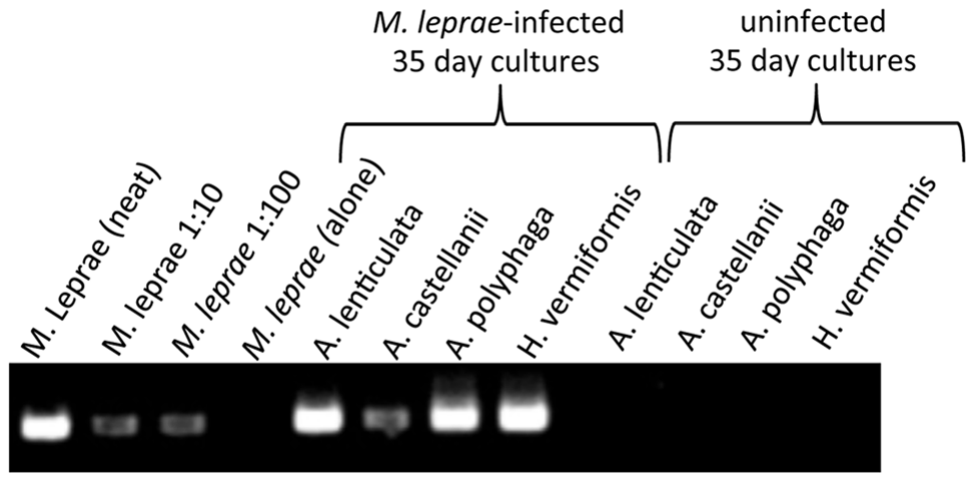
The 129 bp *M. leprae*-specific RLEP sequence is amplified from nucleic acid extracted from both *M. leprae* and 35 day cocultures of *M. leprae* + *A. lenticulata*, *A. castellanii*. *A. polyphaga* or *H. vermiformis* str 172. The amplicon was not apparent from 35-day cultures of axenic *M. leprae* or uninfected cultures.

Finally, to lend further support to the long-term persistence of *M. leprae* bacilli inside amoebic cysts, cocultures of *M. leprae* and encysted amoebae kept for 35 days at 32°C were extracted for nucleic acid and analyzed for the presence of the *M. leprae*-specific 129-bp RLEP amplicon reported to be present at 28 copies per bacterium genome [30]. All *M. leprae*/amoebae cocultures retained a strong RLEP amplicon signal similar to purified *M. leprae* genomic DNA (Fig. 8). Interestingly, in our gel-based PCR analysis, we were unable to detect any RLEP signal from parallel axenic cultures of *M. leprae* in respective amoebae medium indicating a significant degradation of the bacterial chromosome over the 35-day incubation period at 32°C in the absence of amoebae. Altogether, these results suggest that the acid-fast bacilli observed in Figs. 5 and 7 were indeed *M. leprae* that are remaining viable by virtue of being phagocytosed and housed by common FLA.

### Bacilli extracted from encysted and re-emergent cocultures of *A. castellanii* and *A. polyphaga* are capable of growth in nu/nu FP

**Figure 9:**
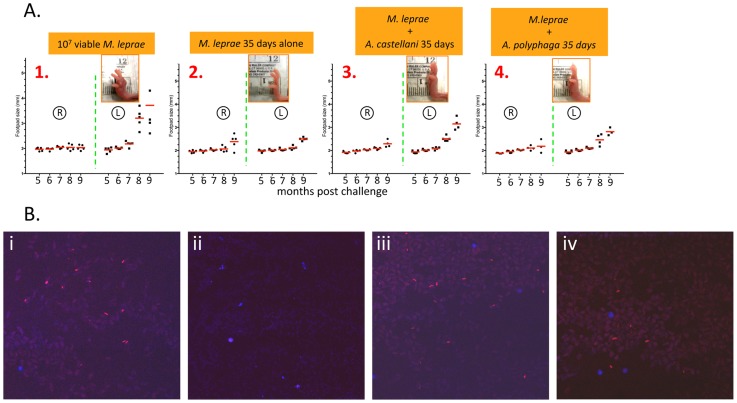
Mice were challenged in the footpad with 10^7^ viable *M. leprae* from *nu/nu* mouse FPs (Panel A, 1), or bacilli extracted from 35 day cultures of *M. leprae* alone in amoebae medium at 32°C (Panel A, 2), bacilli extracted from>30 day cultures with *M. leprae* +*A. castellanii* cysts at 32°C (Panel A, 3) and bacilli extracted from>30 day cultures with *A. polyphaga* cysts for 32°C (Panel A, 4) circled R denotes right unchallenged footpad; circled L denotes the left challenged footpad. Two investigators in a double blind fashion measured footpads and the average thickness was determined and plotted on the graph. The timeframe used to follow the appearance of FP lesions from mice injected with coculture-extracted bacilli was based upon the time after the first injection. Inset pictures in the graphs are photographs of selected left footpads from each experimental group. Panel B shows photomicrographs of material obtained by FNA biopsy of the lesions. Samples were stained with the mycobacteria-specific, auramine/rhodamine and examined under a confocal microscope. Red staining bacilli are indicative of acid-fast organisms. Significant amounts of red-staining bacilli were observed in biopsies derived from the positive control samples (Panel B, i) as well as both amoebae cocultures (Panel B; iii and iv). No acid-fast bacilli were observed in biopsies from footpads challenged with *M. leprae* cultured alone without amoebae (Panel B, ii). Blue staining is DAPI^+^ nuclei fragments from mouse mononuclear cells.

To determine whether the viable bacilli extracted from 35-day amoebae cocultures were capable of causing characteristic *M. leprae*-induced FP indurations in infected mice, bacillary extracts were injected directly into the left FP of athymic *nu*/*nu* mice, and FP pathology was monitored over a period of 8 months. All experimental bacilli that were injected into *nu*/*nu* FPs were extracted from cocultures from either *A. castellanii* or *A. polyphaga* that remained encysted with *M. leprae* bacilli at 32°C in amoebae encystment medium for 33 days followed by excystment for 2 days as described above. The appearance of FP lesions in the *nu*/*nu* mouse model for leprosy is typically very slow with measurable swelling appearing only after 4–5 months post challenge (using an infecting dose of 2–5×10^7^ bacilli/FP)[37]. Since the number of bacilli that were extracted from excysted cultures were considerably lower than the amount extracted directly from FP, we chose to inject FPs with coculture-extracted bacilli every other week for a total of three times in order to decrease the time of emergence of FP symptoms. As positive controls, five mice were challenged in the FP with 10^7^ *M. leprae* bacilli freshly extracted from infected *nu*/*nu* FPs in a manner that is routinely performed to passage *M. leprae* in the laboratory (Fig. 9A, panel 1). Mouse FPs were also injected with *M. leprae* kept in amoebae medium alone for identical periods of temperature and days (Fig. 9A, panel 2). FP swelling was assessed monthly using a Vernier digital caliper and plotted as illustrated in Fig. 9 (Fig. 9A; panels 1–4). During the 6.5 months post-challenge, measurable swelling of the left FP was consistently evident in the positive control animals. In contrast, measurable swelling of the FPs challenged with bacilli extracted from *A. castellanii* and *A. polyphaga* cocultures was not detectable until 7.5–8 months post-challenge but the rates of swelling were similar to early stages of the positive controls (Fig. 9A; compare panels 1 with 3 and 4). There was no detectable FP swelling in animals that were injected with *M. leprae* kept for 35 days at 32°C in amoebae medium alone (Fig. 9A, panel 2; compare photo insets). Any increase in measurement of right FPs or FPs injected with *M. leprae* in medium alone was due to increasing size of the FP because of the overall growth and development of the animal over the duration of the experiment. These results thus indicate that bacilli extracted from long-term (35 days) cocultures of *A. castellanii* and *A. polyphaga* are capable of growth in nu/nu mice FP (albeit with a 2 month delay) similar to *M. leprae* extracted conventionally from donor nu/nu mouse FPs.

### Auramine/rhodamine staining of tissue obtained by (FNA) biopsy of challenged nu/nu footpads followed by PCR analysis indicated the presence of acid-fast bacilli with *M. leprae*-specific amplicons

To show that the swelling measured above contained a considerably high burden of acid-fast bacilli, small samples of tissue were extracted by FNA biopsy from challenged FPs. Smears were stained for subsequent fluorescence microscopic analysis with DAPI for cell nuclei and auramine/rhodamine for acid-fast bacilli. Fluorescence micrographs show considerable (red staining) acid-fast bacilli in FP tissue obtained from all of the mice that were challenged with *M. leprae* derived from freshly harvested passage mice (positive controls) (Fig. 9B; panel i) and in 4 out of 5 of those from each category challenged with bacilli extracted from 35 day cocultures of *A. castellanii* or *A. polyphaga* (Fig. 9B, panels iii and iv). There was no evidence of acid-fast bacilli in FNA-extracted tissue from mice FPs challenged with *M. leprae* from axenic cultures without amoebae (Fig. 9B, panel ii). These results suggest further that FP lesions in these mice were the direct result of viable *M. leprae* extracted from long-term amoebic cocultures.

**Figure 10:**
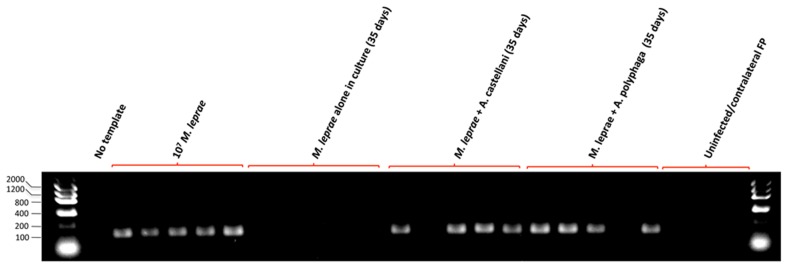
Tissue extracted from *nu/nu* mouse footpads by FNA 7 months after challenge either with 10^7^ bacilli from passage mice (“10^7^ *M.leprae*”) or extracted from of 35 day cocultures of *M. leprae* with either *A. castellanii* or *A. polyphaga* indicate 80% (4 out of 5) positive PCR signals for the *M. leprae-*specific repetitive element (RLEP). The *M. leprae* specific amplicon was not detected in FNA material from mice challenged with *M. leprae* kept in amoebae medium alone.

To further confirm that the acid-fast bacilli observed in tissue was indeed *M. leprae*, nucleic acid was extracted and tested in PCR analysis for amplification of the *M. leprae*-specific RLEP sequence as in Fig. 8 above. Positive PCR signals were obtained from FNA tissue from all mice challenged with *M. leprae* directly from passage mouse FPs and 4 out of 5 of the mice challenged with bacilli extracted from cocultures of *A. castellanii* or *A. polyphaga* (Fig. 10). The one mouse FP in each group that was negative by PCR was also negative by auramine-rhodamine staining for acid-fast bacteria. By contrast, after 8 months post-challenge, there was no evidence of the *M. leprae* RLEP PCR signal in any of the FPs from mice challenged with *M. leprae* maintained in axenic cultures of amoebae medium. PCR analysis of FNA tissue for amplification of *Acanthamoeba*-specific 18S rRNA sequences [38] was negative as well, suggesting that either the extraction method for obtaining the bacilli effectively killed the amoebae or the mice successfully resolved any residual amoebae infection over the long-term experimental period. This confirms that the acid-fast bacilli observed in FP lesions produced by challenge with bacilli extracted from amoebae are most likely *M. leprae* and that the bacilli remain viable and capable of transmitting FP pathology for up to 35 days in cocultures of two different *Acanthamoeba* spp.

**Figure 11:**
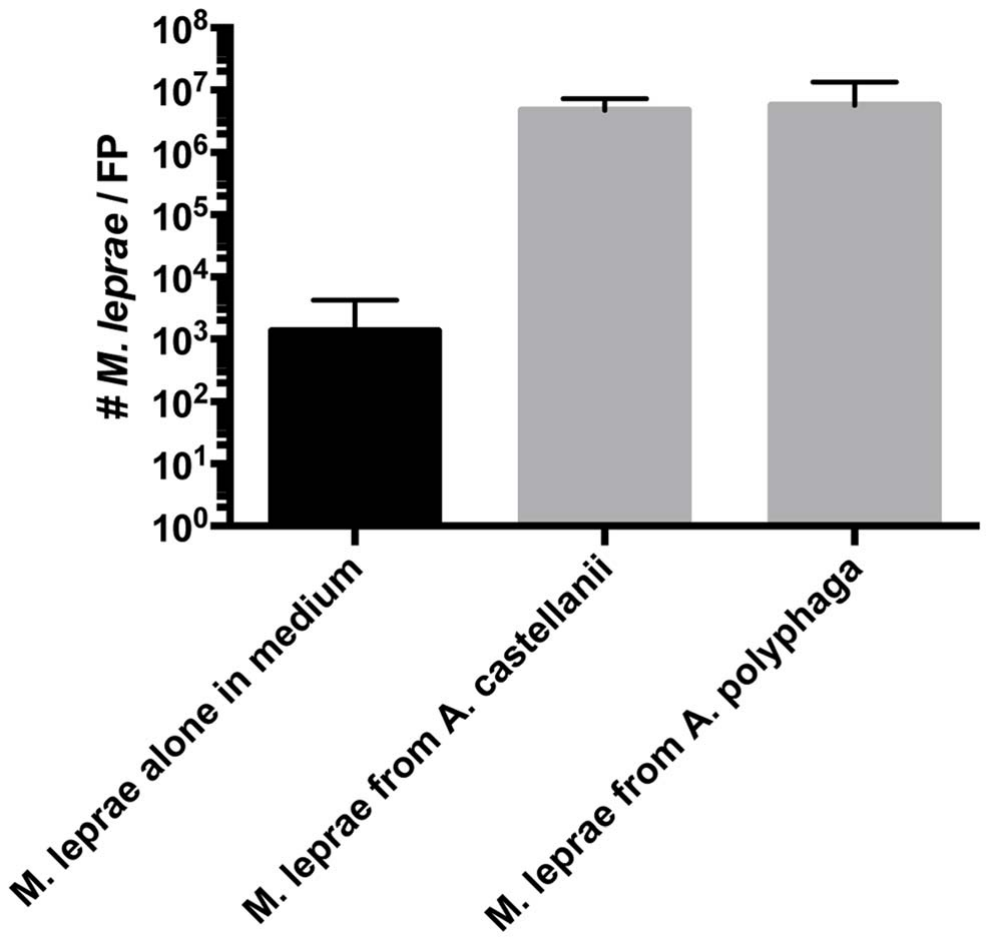
Comparative enumeration and quantification of *M. leprae* in mouse FPs that were either challenged with *M. leprae* collected from 35 day culture medium alone (PYG) or *M. leprae* extracted from 35 day cocultures with *A. castellanii* or *A. polyphaga*. There was a 3–3.5 log increase in the number of bacilli recovered from FPs challenged with *M. leprae* extracted from amoebae coculture when compared to the amount detected from FPs challenged with *M. leprae* maintained in amoebae medium alone for 35 days. Error bars indicate the Mean and Standard Error Measurements (SEM) of 5 mice per group.

The observation that there is a considerable increase in the number of acid-fast bacilli in FPs (Fig. 9) coupled with the strong PCR signal obtained from primers for the *M. leprae*-specific RLEP (Fig. 10), strongly suggested the presence and growth of *M. leprae* in FPs challenged with amoebae-derived bacteria. However, to confirm that the 35 day-amoebae cocultured *M. leprae* are indeed capable of replication within the FP, we performed a TaqMan quantitative PCR analysis for the RLEP region of *M. leprae* to compared the number of *M. leprae* present in FPs challenged with bacilli derived from amoebic cocultures vs. the number from FPs challenged with *M. leprae* grown alone for 35 days in amoebae medium. Fig. 11 shows the results of the Taqman analysis. The amount of *M. leprae* extracted from FP challenged with coculture-derived bacilli exhibited a significant increase of the RLEP signal when compared to those challenged with *M. leprae* kept in medium for 35 days. The data represents a 3–3.5 log increase *M. leprae* in FPs challenged with *M. leprae* from amoebae cocultures compared to those challenged with *M. leprae* maintained in axenic cultures medium. These data confirm that *M. leprae* extracted from 35 day encysted amoebae cultures are capable of replication in the *nu/nu* mouse footpad.

## Discussion

The precise manner in which leprosy is transmitted is unknown. Until recently it was widely believed that the disease was transmitted by proximal contact between untreated or asymptomatic cases of leprosy and healthy people. Currently, the possibility of transmission by the respiratory aerosol route has gained considerable interest [39]. Other means such as transmission through insects [40] [41] has been considered but there has not been any substantial evidence supporting that claim. The possibility of discharge of *M. leprae* from the nasal mucosa begs the question of how the discharged organism remains viable in between hosts. Since *M. leprae* is a fastidiously obligate intracellular bacterium, it would be reasonable to assume that it could find safe refuge in the environment by interaction with ubiquitous free-living organisms with physiological semblance to human phagocytes. Recently, it was shown that *M. leprae* could be taken up by FLA, survive and remain viable intracellularly in these protozoa for a period of at least 72 hr [42]. In the current study, we demonstrate that *M. leprae* can survive and remain virulent for at least 35 days within amoebal cysts from both *A. castellanii* and *A. polyphaga* as determined by their ability to transfer infection to recipient *nu*/*nu* mouse FPs. Furthermore, we show that acid-fast bacilli extracted from *M. leprae*/amoebae cocultures with *A. lenticulata*, *A. castellanii*, *A. polyphaga*, *H. vermiformis* str. ATCC 50237 and *H. vermiformis* str. 172 remain viable for over 8 months in encysted amoebae as determined by viability staining of bacilli *in situ* within cysts or from the those extracted from the cysts. These data provide a proof of concept that *M. leprae* can be phagocytized and lysosomally occupy common environmental FLA trophozoites, survive encystment while remaining viable and are fully capable of infectivity under suboptimal conditions endured by the amoebic cyst. Although *M. leprae* has been shown to be approximately 30% viable in terms of membrane integrity by BacLight after two weeks in optimized medium [36], survival in either amoebae medium described here is very detrimental to axenic *M. leprae* and necessitates refuge within amoebae. It can be reasonably argued that possible environmental reservoirs for this fastidious bacillus are common FLA.

*M. leprae* cannot be cultured, and therefore there are limited means available to ascertain its viability in long-term amoebae culture. No single method of investigation can be utilized to confirm its viability in amoebic cysts. For example, the specificity of serological techniques and PCR can be impaired by antigenic cross-reactivity and PCR contamination, respectively. Therefore, we embarked on an exhaustive set of experiments in order to be absolutely certain that *M. leprae* can be engulfed and remain viable within the amoebae examined and to be confident that the acid-fast organisms detected in FPs from mice challenged thereof were indeed *M. leprae* from amoebae cocultures. We built upon this conclusion by first demonstrating that *M. leprae* is phagocytized by amoebae as determined by microscopic and flow cytometric analysis revealing that optimal uptake requires active temperature-dependent metabolism and viability of both organisms. Subsequently, we assessed bacillary viability by use of a two-color, Syto9/propidium iodide fluorescence staining that scores for membrane damage in individual bacilli and is a proven technique that correlates well with other viability measures for *M. leprae* such as radiorespirometry [36]. Virtually all of the bacilli extracted from long-term cocultures at 32°C were propidium iodide negative (PI^-^) and Syto9^+^ indicative of viable organisms. Control cultures containing *M. leprae* alone in amoebae medium showed considerable degradation of the bacilli that was virtually 100% propidium iodide positive after two weeks and were undetectable after 35 days at 32°C (Fig. 5). We also observed occupancy of acid-fast organisms within amoebal cysts for 8 months post culturing. The extracted bacilli emerged either as intact extracellular bacilli or residents of acid-rich organelles of recently excysted trophozoites (Figs. 5 and 7). Most emergent bacilli were deemed viable by Syto9^+^/propidium iodide negative staining. PCR analysis of nucleic acid from encysted cocultures amplified the *M. leprae*-specific RLEP element strongly supporting the fact that the acid-fast bodies observed in cysts were indeed *M. leprae*. The loss of RLEP PCR signal in 35 day axenic *M. leprae* cultures is curious since *M. leprae* DNA has been known to persist in tissues for very long times after host death. The loss of signal in these cultures is likely due to release of soluble nucleic acid from the dead bacilli in the liquid medium that is lost when washing. In addition, detection of RLEP in archeological samples is performed using the more sensitive TaqMan PCR methodology. Most importantly, *M. leprae* survival and retention of virulence in cocultures were confirmed by transference of extracted bacilli from 35-day cocultures of *A. castellanii* and *A. polyphaga* into *nu*/*nu* mouse FPs. 80% of the mice (4 out of 5) challenged with either coculture developed FP swelling with histological evidence of acid-fast bacilli. Collectively, these data confirm that *M. leprae* can indeed survive for extended periods of time in encysted FLA cultures and is capable of growth in *nu/nu* mice FP.

The number of viable *M. leprae* extracted from these cocultures was significantly less than the initial number used to infect the trophozoites. 1.5×10^7^ bacilli were used to infect 3×10^6^ (MOI  = 5) amoebae and, based on microscopic field counts, the estimated number of *M. leprae* harvested from amoebic cysts and injected into FPs was between 10^5^–10^6^ per injection. This may be due to several reasons: i) Only approximately 30% of the trophozotic amoebae were observed to be capable of encystment as the shift in the transcriptional program necessary for this transformation is considerably complicated and incompletely understood [35]. Since incomplete transformation to cysts might impose a restriction on the actual numbers of bacilli housed therein there would be a considerable culling and reduction in the numbers of protected and viable *M. leprae*. ii) To facilitate processing, bacilli were extracted from cysts that were first induced to excyst. Studies have shown that bacteria residing in *Acanthamoeba* cysts are generally housed both within the cytoplasm as well as within the cyst walls between the endo- and ectocyst shells as is the case for *Acanthamoeba* spp. [35]. The extrusion process of emerging trophozoites from cyst wall pores known as ostioles has the potential of leaving a considerable number of bacilli in the cyst wall remnants that may be either unavailable for infection or are pelleted in the slow speed centrifugation steps used in the purification of the extracted bacilli [43]. iii) The extraction process of recently emergent trophozoites in this study involved treatment with SDS. Due to their unique cell wall, mycobacteria can survive relatively long exposures to detergents [44] but the effect of SDS treatment on long-term viability of *M. leprae* has, to our knowledge, not been determined and may be a factor in reduced viability of extracted bacilli. However, there was no indication of membrane damage in extracted bacilli as assessed by viability staining. iv) There may be a slow loss of viability over time in the amoebae cyst cocultures if the cultures are unable to optimally support the bacilli and the cysts are simply “buying time” for *M. leprae*. Also it is possible that not all the extracellular bacteria were capable of being endocytosed by the amoeba. It has been shown that the inevitable clumping that occurs in cell-free *Mycobacteria* suspensions contain aggregates that are not efficiently taken up by either amoebae or macrophages [11], [16], [45]. Regardless, the encystment of the bacilli prolongs viability empirically. Molecular enumeration, and analysis of viability transcripts by reverse transcriptase-based quantitative real-time PCR will likely provide some insight into the longevity of the bacteria in cysts. Recently, transcripts encoding the *M. leprae*-specific ESAT-6, heat-shock protein 18, superoxide dismutase A and the 16S rRNA subunit have been determined to be sensitive viability indicators for *M. leprae* [28], [34]. Many of these approaches are planned in future endeavors.

Prior observations that amoebae can house and transport *L. pneumophila* and can serve to increase the virulence of *M. avium* have raised concern that protozoa have the potential to be general environmental reservoirs or vectors of human pathogens [11], [45]. It has been considered that adaptation of organisms to parasitism, commensalism or simply endocytobiontism of FLA might have molded environmental microorganisms to infect and persist in human phagocytes. That is to say, the process of the selection of environmental microorganisms for resistance to digestion by predatory FLA behaving as feral macrophages might be a driving force in the evolution of pathogenic environmental bacteria. Such a process may likely be the “missing link” between ecology and pathology [46]-[48]. FLA are present worldwide [49] and have been isolated from soil [50]–[53], water [54]–[58], air [59], and the nasal mucosa of otherwise healthy human volunteers [60]–[62]. The fact that there are repeated observations of clinical leprosy in those that appear to have no history of exposure to known cases [63]–[66] and that leprosy tends to cluster in areas proximal to water sources [67], [68] strongly suggest that *M. leprae* has extra-human environmental sources [69], [70] and those environs are also compatible with the globally ubiquitous FLA.

The natural environmental landscape for amoebae (as is for most organisms) is not static and, as such, various adaptive genetic programs have evolved to survive dynamic and potentially detrimental conditions. Exposure to suboptimal conditions such as starvation, extreme temperatures, excessive UV light, radiation, pH changes, as well as exposure to biocides induce amoebae trophozoites to undergo encystation [16]. Amoebae exist in the environment cyclically transforming from free-feeding trophozoites to highly dispersible and resilient cysts. *M. leprae*, by virtue of having a slow generation time can likely withstand the confines of the amoebal cyst allowing bacillary viability to persist longer in this manner. Moreover, the coculture of *M. leprae* with *Acanthamoeba* or *Hartmannella* spp. is particularly suited for both bacteria and amoebae since their temperature optima are compatible for both initial infection of trophozoite and long-term “storage” of cysts.

It has been shown that *M. leprae* can remain viable if lyophilized in the presence of 10% skim milk-water [71]. In addition, viability was preserved up to 4 years at 4°C. This work also demonstrated clearly that the composition of the solution for suspending the bacilli was critical for the maintenance of *M. leprae* viability by lyophilization—with skim milk being 100-fold more effective that water or water with 10% fetal calf serum. With respect to viability in amoebic cysts, these results are intriguing. While desiccation may provide a means of survival/viability to the bacillus, it is unlikely that drying per se is a “natural” means of persistence since most if not all of the remaining endemic areas are those of high humidity and abundant water. The amoebic cyst (in particular the acanthamoeba cyst) is a very efficient desiccant that is essentially devoid of water. The natural "arid" environment inside of cysts allows long-term survival (years) of the amoebae in the face of drought etc. by virtue of its impermeable cellulose wall [35]. Could the same mechanisms (dehydration etc.) that provides viability to the amoebae be "hijacked" by the captured *M. leprae* to provide long-term viability to the bacillus? Future experimentation will likely reveal answers to these rather intriguing questions.

It would be intriguing to determine whether *M. leprae*, by virtue of residing in cysts, has evolved its own dormancy program in order to persist and maintain or enhance viability or virulence. Also, active prompting of protozoan encystment by bacteria has thus far only been demonstrated for *L. monocytogenes* suggesting that these bacteria have a selective advantage of exploiting the cysts' ability to serve as vehicles and to assume dormant stages that aid dispersal in the environment [48], [72]. Whether this is the case for *M. leprae* as well awaits further investigation. Other outstanding questions include the determination of (A) whether *M. leprae*, by virtue of being transmitted via amoebae, can enter host macrophages via a Trojan horse mechanism thereby changing the overall pathogen-associated molecular pattern (PAMP) presented to innate immune cells and subsequently altering innate and adaptive responses to the benefit of the pathogen; (B) whether *M. leprae* is capable of multiplying within amoebae or is simply maintaining survival therein. The results described in this current work do not demonstrate that *M. leprae* is capable of multiplying inside amoebae but are suggestive of a role for FLA providing sustenance to maintain viability of the bacilli; (C) whether the leprosy bacillus requires periodic excystment as is likely the case in the natural world in order to re-infect emergent trophozoites or human host cells; and, finally (D) whether the virulence of *M. leprae* is affected either positively or negatively by its passage through amoebae. Future experimentation including testing for appearance of disease by transferring *M. leprae* from our other existing cocultures of *A. lenticulata* and *H. vermiformis* strains to *nu/nu* mouse FPs and determining whether mice challenged directly with *M. leprae*-infected amoebae (cysts or trophozoites) display any differences in progress to disease should resolve some of these issues.

In summary, we show that *M. leprae* is capable of prolonged survival in three common and ubiquitous species of *Acanthamoeba* and two strains of *Hartmannella*. At this point we are unsure of whether this endocytobiotic relationship in nature serves to allow some FLA to function as transmission vehicles/vectors, a Trojan horse and/or biological reservoirs for *M. leprae*. It will be fascinating to determine whether FLA in general provide an environmental sanctuary possibly facilitating virulence and contributing to microbial survival in harsh conditions along with aiding transmission to susceptible hosts. Future experimentation will clearly unravel these issues.

## Supporting Information

S1 Fig Axenic cultures of *A. lenticulata*, *A. castellani*, *A. polyphaga*, *H. vermiformis* str. ATCCand *H*. *vermiformis* str. 172 were infected with *M. leprae* isolated from either nu/nu footpads or armadillo tissue at various M.O.I. (1∶100 (black diamonds), 1∶50 (black inverted triangles), 1∶10 (black upright triangles), 1∶5 (black squares) and 1∶1 (black circles) [amoebae∶*M.lepra*e]) in 1/10 PYG at either 32°C or 4°C. Aliquots were taken at the time of infection and each hr after 2 hrs of incubation and analyzed by flow cytometry. Prior to flow cytometric analysis the aliquots were centrifuged 3X at 600 X g to pellet and remove any cell-free bacilli. Samples were analyzed by flow cytometry and the mean fluorescence intensity was plotted per unit time in culture.
(TIF)
