# Supplementary figures and images for: Long-term Survival and Virulence of Mycobacterium leprae in Amoebal Cysts
Source: PLoS Negl Trop Dis. 2014 Dec 18;8(12):e3405. doi: 10.1371/journal.pntd.0003405 (PMC4270725; doi:10.1371/journal.pntd.0003405)

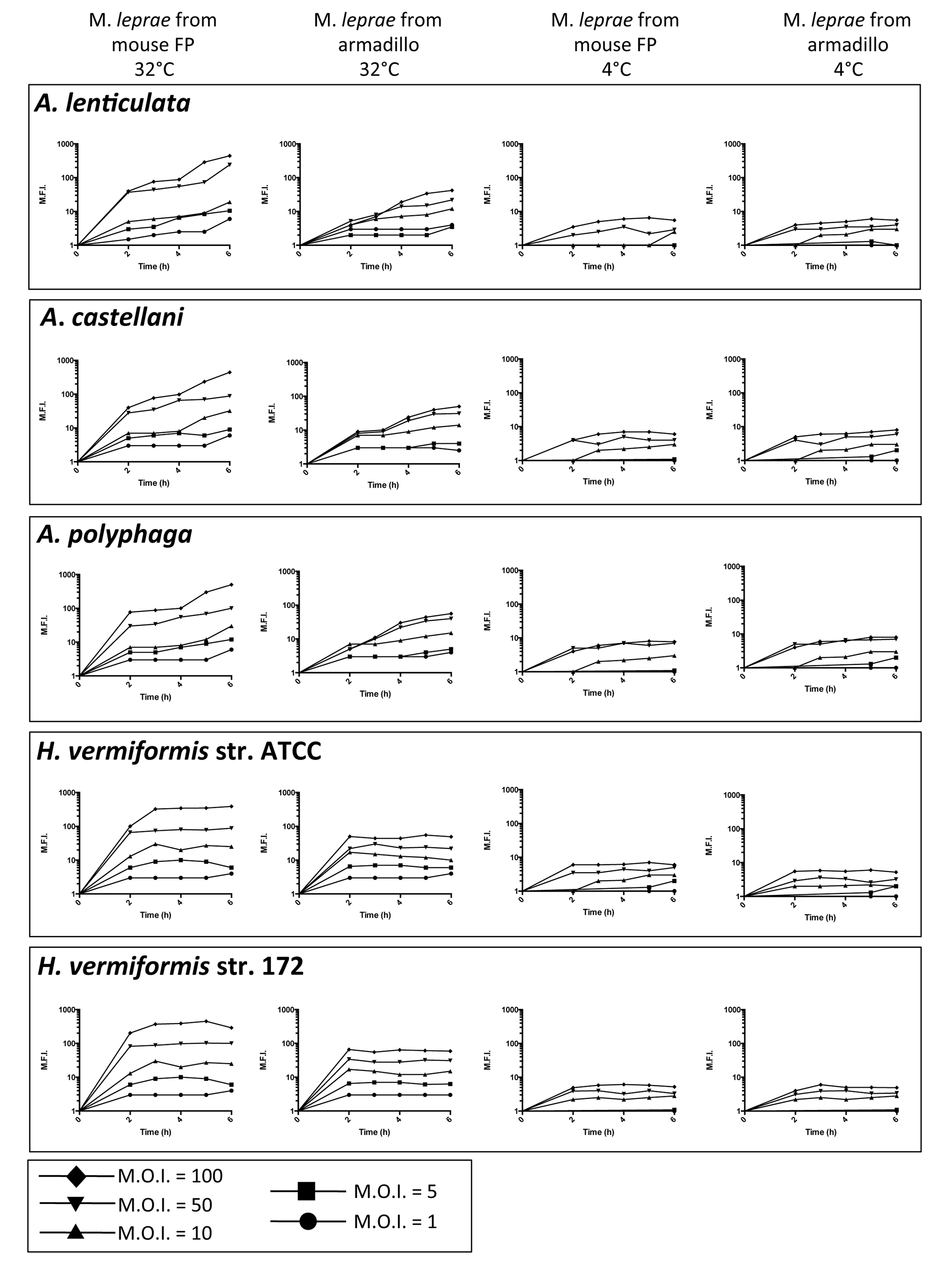

Supplement: S1 Fig — Axenic cultures of A. lenticulata, A. castellani, A. polyphaga, H. vermiformis str. ATCCand H. vermiformis str. 172 were infected with M. leprae isolated from either nu/nu footpads or armadillo tissue at various M.O.I. (1∶100 (black diamonds), 1∶50 (black inverted triangles), 1∶10 (black upright triangles), 1∶5 (black squares) and 1∶1 (black circles) [amoebae∶M.leprae]) in 1/10 PYG at either 32°C or 4°C. Aliquots were taken at the time of infection and each hr after 2 hrs of incubation and analyzed by flow cytometry. Prior to flow cytometric analysis the aliquots were centrifuged 3X at 600 X g to pellet and remove any cell-free bacilli. Samples were analyzed by flow cytometry and the mean fluorescence intensity was plotted per unit time in culture. (TIF) [file pntd.0003405.s001.tif]
